# Supplementary material for: Longitudinal imaging of Caenorhabditis elegans in a microfabricated device reveals variation in behavioral decline during aging
Source: eLife. 2017 May 31;6:e26652. doi: 10.7554/eLife.26652 (PMC5484621; doi:10.7554/eLife.26652)
Supplement: Supplementary file 1. — DOI: http://dx.doi.org/10.7554/eLife.26652.027 [file elife-26652-supp1.pdf]

WorMotel Protocol

Fang-Yen Laboratory, University of Pennsylvania

Last Update: Jan 2017

Email: fangyen@seas.upenn.edu

Introduction

We have developed a multi-well imaging platform for parallel monitoring of *C. elegans*, each confined to an individual well. These 'WorMotel' devices are constructed using polydimethylsiloxane (PDMS) cast from a photopolymer 3D-printed mold. Each device contains an array of 3-mm-diameter wells optimized for worm cultivation and imaging. Each well is filled with NGM agar and seeded with food bacteria before adding a worm.

Reagents

1. Modified NGM media prepared by the same protocol as standard NGM agar, except agar is replaced with a low gelling temperature agarose (A20070, Research Products International Corp.). Streptomycin (optional) can be added at a final concentration of 100 µg/mL to minimize bacterial contamination. The agarose solution is then kept at 50°C in a heat block before use. Standard NGM agar can also be used, but then a separate pipette is usually needed to fill each well due to rapid gelling of agar.

2. To avoid progeny, FUdR can be added to a final concentration of 200 µM. FUdR degrades at temperatures above 40°C, so add it to the agarose solution just before filling the WorMotel.

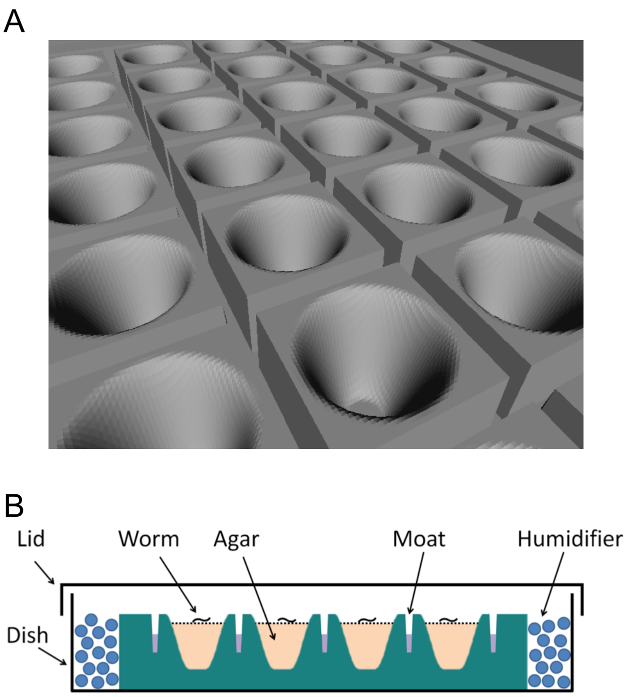

Fig. 1. (a) 3D rendering of the WorMotel design. (b) schematic cross section including agar, moats, humidifying crystals, and worms

3. OP50 or DA837 (or other food bacteria) suspension. The WorMotel is also compatible with standard protocols for feeding RNAi.

4. Moat solution: 100 mM copper sulfate. This is optional to reduce escape rate. Copper sulfate moat does not affect N2 lifespan or developmental rate.

5. OmniTray (Nunc) or 10 cm petri dish to form a humidified chamber.

6. To maintain the humidity of the wells, use water absorbing crystals (AgSAP polyacrylamide water absorbing crystals, 200-800 microns, M2 Polymers) to sterile water at a mass ratio of

about 1:170. The ratio can be adjusted to increase or decrease humidification. For short-term experiments, wet Kimwipes are also suitable.

7. For long-term experiments, Parafilm should be used to seal the edges of the dish.
8. A 20% solution by weight of Tween 20 in water is used as an anti-fog coating on the inside of the chamber lid.

WorMotel preparation Protocol

WorMotel sizes

For high resolution behavior measurements, we use a 48-well (6 x 8) array. For assays requiring a larger N, we use a 240-well (12 x 20) array designed to fit within an OmniTray (Nunc), which has the same external dimensions as a microplate.

The distance between centers of wells is 4.5 mm, following the specifications of a 384-well microplate.

Molding WorMotel from master

The WorMotel chips are made by molding PDMS by a 3D-printed acrylic photopolymer mold. To fabricate a chip, mix PDMS (Dow Corning Sylgard 184 Silicone Encapsulant, e. g. from Ellsworth Adhesives) according to manufacturer specifications at a 10:1 ratio of base material to cross linker in a 50 ml conical tube or other disposable container. Pour PDMS into the mold. Remove bubbles by placing under vacuum (e.g. vacuum dessicator or vacuum oven without heat) for 30-60 min. If there are a few remaining bubbles they can be popped manually with a pipette tip.

To cure PDMS, place at 37-40C for 12 hr. Curing at temperatures higher than 40 C will make it difficult to de-mold PDMS.

Peel PDMS from the mold using a metal spatula, taking care not to crack the mold.

Sterilization

Sterilize the WorMotel chip by autoclaving or boiling in water for 5 minutes. Dry the chip by stamping excess liquid onto a paper towel or by letting sit overnight in a clean hood. Chips can be reused many times. Boiling is effective for removing residue from previous experiments.

Plasma Cleaning or surfactant

Best results are obtained if a WorMotel undergoes plasma cleaning after drying and prior to filling. Plasma cleaning renders the PDMS temporarily hydrophilic, facilitating the loading process and helping to create flat agar surfaces. Plasma cleaners are common in many microfabrication and microfluidics labs.

We use a 4-minute oxygen plasma treatment on a Plasma Etch PE-50 at an oxygen flow rate of 5-10 SCCM, or a 30 s – 1 min oxygen plasma treatment on a Plasma Preen II machine. After this treatment, the chip maintains adequate hydrophilicity for about 1 hour.

An acceptable alternative to plasma cleaning is to add 0.003% (v/v) Tween 20 or Tween 80 to the agar prior to filling. Tween, a biocompatible surfactant, reduces the contact angle between the liquid agar and PDMS in a manner similar to plasma cleaning. We have not observed any differences in behavior or lifespan at this concentration of Tween.

Humidification

To maintain humidity inside the dishes, we use water-absorbing crystals (purchased on Amazon). Sterile water is added to the crystals in a ratio of 150:1 water:crystals by weight. Add 15-20 g of wet water-absorbing crystals around the WorMotel to maintain humidity. To minimize dehydration, Parafilm plates or monitor the weight of dishes and add water every day to compensate for evaporation.

Moat loading

Pipette 100 mM copper sulfate repellent solution into the moat at a corner, giving a liquid depth of ~1 mm in the moats. With sufficient plasma cleaning, the solution should readily wet the hydrophilic surface and propagate through the moat, leaving little or no liquid above the surface. If working without plasma cleaning, adding a surfactant such as Tween will help with moat loading.

NGM buffer (same recipe as NGM agar but without agar, peptone, or cholesterol) can also be used to fill the moat. Although both copper sulfate solution and NGM can prevent escaped worms from entering other wells, our results showed that the copper sulfate solution yields a significantly reduced rate of escape over 6 days (5%) compared to NGM moat (25%).

Some researchers have reported good results without using a moat solution.

Well filling

To fill the wells, approximately 15 µL of modified NGM agar (prepared as described above) is transferred into each well with a P200 pipette or multichannel pipette. Try to avoid bubbles or nicks in the agar due to the pipette.

If the agar does not fill the well properly, remove agar by pipette or vacuum aspirator and refill.

Seeding

After filling with agar, bacteria suspension is transferred to the agar surface and allowed to dry. Avoid touching the agar surface with the pipette(s) as this can create gashes that worms can crawl into. For lifespan experiments we spin down bacteria in LB cultures in a 50 mL tube, resuspend in water + NaCl, spin down again, remove supernatant, and add 5 µL of concentrated suspension to each well.

For experiments with larvae we recommend adding only 1 µL of bacteria solution. This yields a thinner lawn and facilitates visualization of younger worms which are smaller, thinner, and more transparent.

Adding worms

With a worm pick, glass mouth pipette, or low-retention pipet, transfer individual animals to each well. When using a worm pick, this process is facilitated when the well surface is still partially wet with seed bacteria.

Anti-fog coating

To prevent water condensation on the lid during imaging, the lid of the petri dish or the OmniTray is covered with a thin layer of surfactant. Spread a 20% Tween 20 solution on the inside of the lid, pour off the excess liquid, and allow the lid to dry in a sterile hood before placing it on the petri dish or OmniTray. If there is not enough Tween on the inside of the lid condensation can occur, especially after 2 or 3 weeks. In this case, simply remove the WorMotel from the imaging rig, reapply Tween to the inside of the lid, and resume imaging. Be sure to cover the WorMotel plate with a spare lid while waiting for the Tween to dry to prevent agar desiccation. If present, condensation drastically interferes with image quality and can make it difficult to measure lifespan using image analysis software.

Note: Temperature fluctuations are the most common cause of condensation. Therefore, it is important that WorMotel experiments be conducted in a constant temperature environment. Condensation can make it impossible to distinguish between worm movements and water droplet shifts on the plate lid. Avoiding condensation is the most important step in acquiring reliable data. If condensation does occur, it can be removed by removing the plate from the imaging station and reapplying Tween to the lid. Images where condensation was present should be censored from the analysis.

Preventing contamination

To minimize contamination, preparation can be done on a clean bench containing a dissecting microscope. Media can be supplemented with antibiotics (Streptomycin) and/or antifungal (Nystatin) agents to reduce contamination.

Imaging system

Almost any microscope with a camera attachment and an appropriate field of view can be used to acquire behavioral imaging data. We use a custom-designed imaging rig described in Churgin and Fang-Yen, “An imaging system for C. elegans behavior”, in C. elegans, Methods and Applications, G. Haspel and D. Biron, eds., Methods in Molecular Biology 1327: 199-207 (2015) (available on the Fang-Yen lab web page)

The WorMotel was designed to be used with dark field imaging. We use red LED strips (Oznium.com) for illumination, 5” long, qty 4, arranged into a square below the Imaging patform.

Many types of cameras will work for behavioral imaging. We primarily use 5MP cameras from The Imaging Source with IC Capture software.

Data analysis

We perform analysis using custom scripts written in MATLAB. Software is available upon request.
