# Supplementary material for: Longitudinal imaging of Caenorhabditis elegans in a microfabricated device reveals variation in behavioral decline during aging
Source: eLife. 2017 May 31;6:e26652. doi: 10.7554/eLife.26652 (PMC5484621; doi:10.7554/eLife.26652)
Supplement: Supplementary file 4. — DOI: http://dx.doi.org/10.7554/eLife.26652.030 [file elife-26652-supp4.zip › Imaging_and_stimulation_materials/LightStimulation_Readme.rtf]

Light Stimulation Read Meby Matt ChurginFang-Yen Lab, University of PennsylvaniaLast updated 13 Mar 2017This ReadMe instructs how to use Matlab to control LEDs using an I/O device and a solid state relay.1.  Run “Light_stimulation_control_170313.m”2.  The code supports using either a Labjack or NIDaq to control the LEDs.  If you are using a Labjack, use the first three cells of the m-file.  If you are using a NIDaq, use cells 4 through 6.3. Ensure your LEDs, solid state relay, and power supply are properly connected and turn the power supply on.  Be sure to keep the LEDs facing away from your eyes.4.  Run the first cell to initialize your device.5.  Now you will test your LEDs to ensure the relay and I/O device are working properly.  In the second cell, set Switch1 = 4.5, then run the cell.  The LEDs should turn on.  You can adjust their brightness using the current knob on your power supply.  Set Switch1=0, and run the cell again.  The LEDs should turn off.6.  The third cell is the main program.  The code will turn the lights on twice daily for the amount of seconds specified in “ledontime.”  The default is 10 seconds.  You may set the times at which the LEDs will turn on in “ontimes.”  Run the code and let it run continuously.
