# Supplementary material for: Longitudinal imaging of Caenorhabditis elegans in a microfabricated device reveals variation in behavioral decline during aging
Source: eLife. 2017 May 31;6:e26652. doi: 10.7554/eLife.26652 (PMC5484621; doi:10.7554/eLife.26652)
Supplement: Supplementary file 5. — DOI: http://dx.doi.org/10.7554/eLife.26652.031 [file elife-26652-supp5.zip › WorMotel_LightStim_ProcessingCode/WM_LightStimulation_ReadMe.rtf]

WorMotel with Light StimulationImage Processing Suite Read Meby Matt ChurginFang-Yen Lab, University of PennsylvaniaLast updated 13 Mar 2017Note:  These analysis codes assume images of type ‘png.’  The codes also assume the presence of a periodic light stimulus and non-continuous imaging (i.e. images acquired only surrounding a light stimulus).  More flexibility is forthcoming.Before beginning image processing, ensure that the following files are in the Matlab path, as all these files are required to successfully process the images (as described in Part 1 below):ComputeActivityMatt.mComputeTimeDiffBtwTwoDateVectorsMatt.mGetImageTimeArrayMatt.mmainFile.mROIDefine.mSearchActiveImagingPeriodsMatt.mSearchStimulationTimeMatt.mPart 1:  Image Processing1.  Define Well Centers and Well Size (ROI selection)Run “mainFile.m”Select the first image of the experiment you want to process.Select the default parameters when the next dialog box pops up.You will next be asked to define the grid of WorMotel well centers and the size of a representative well.To select the well centers, click, in succession, on the center of the upper left, upper right, lower left, then lower right well of the WorMotel.  This action define the the grid of well centers.Then, in a single representative well (near the center of the plate is a good choice), click on the upper left and lower right of the well.  This action defines the size of each well.When finished, click on the right side of the image figure in the gray region outside the image.  This concludes ROI selection.  Continue to step 2.2.  Process the imagesSelect the folder with the images you want to process.Depending on how many images you have acquired, this step may take up to a few hours.Progress on the processing of each day’s images should appear in the Matlab command window.When complete, the processed data files (“pdata”) will be located in a folder entitled “Analysis” that resides inside the same folder as where the original images are located.  Inside the “Analysis” folder should be the “pdata” files.  These files contain the activity information for each light stimulation period.Part 2:  Data Analysis, Lifespan Calculation, and Graphing3.  Analyze the processed image dataRun “lifespanCalc_161026.m”Note:  This code assumes there are four experimental conditions.  The conditions are broken up on the plate as rows 1-3, 4-6, 7-9, and 10-12.Select the folder containing the processed data (“pdata”) files.Select all the pdata files and click open.Follow the command prompts.The data will be saved in both .mat format (matlab compatible) and .csv format (Excel compatible).The following .csv files are produced: ‘BaselineActivity’ contains four-minute average activity data for worms prior to each light stimulation.‘StimulatedActivity’ contains four-minute average activity data for worms after each light stimulation.‘Lifespans’ contains the lifespan of each worm for each of four experimental conditions.‘LSCurve’ contains the lifespan curves for each of four experimental conditions.  The lifespan curves can be plotted in using Excel.4. Create lifespan heat mapsRun “lifespanMap.m”This code will produce lifespan activity heat maps for each experimental conditionSelect the folder with the saved .mat file from step 3Select the .mat fileThe code will produce four heat maps that show activity as well as lifespan for each worm, sorted by lifespan.
